# Dielectric Properties of Fluorinated Aromatic Polyimide Films with Rigid Polymer Backbones

**DOI:** 10.3390/polym14030649

**Published:** 2022-02-08

**Authors:** Jian-Jun He, Hai-Xia Yang, Feng Zheng, Shi-Yong Yang

**Affiliations:** 1 Key Laboratory of Science and Technology on High-Tech Polymer Materials, Institute of Chemistry, Chinese Academy of Sciences, Beijing 100190, China; hejianjun@iccas.ac.cn (J.-J.H.); shiyang@iccas.ac.cn (S.-Y.Y.); 2 School of Chemical Engineering, University of Chinese Academy of Sciences, Beijing 100049, China; 3 School of Chemical Science and Engineering, Tongji University, Shanghai 200092, China

**Keywords:** fluorinated aromatic polyimide, low dielectric loss, molecular chain orientation, molecular dynamics simulation

## Abstract

Fluorinated aromatic polyimide (FAPI) films with rigid polymer backbones have been prepared by chemical imidization approach. The polyimide films exhibited excellent mechanical properties including elastic modulus of up to 8.4 GPa and tensile strength of up to 326.7 MPa, and outstanding thermal stability including glass transition temperature (*T*_g_) of 346.3–351.6 °C and thermal decomposition temperature in air (*T*_d5_) of 544.1–612.3 °C, as well as high colorless transmittance of >81.2% at 500 nm. Moreover, the polyimide films showed stable dielectric constant and low dielectric loss at 10–60 GHz, attributed to the close packing of rigid polymer backbones that limited the deflection of the dipole in the electric field. Molecular dynamics simulation was also established to describe the relationship of molecular structure and dielectric loss.

## 1. Introduction

Aromatic polyimide films have been widely used in the microelectronic manufacturing and packaging industry due to its excellent thermal stability, combined mechanical properties, dimensional stability, chemical resistance and electrical insulating properties [1,2,3,4]. With the rapid advancement of communication technology, much attention has been focused on lowering the dielectric loss of polyimide films at high frequency.

It is an effective way to reduce the dielectric constant of the polymer by decreasing the molar polarizability or increasing the molar volume of the molecules. Hence, the dielectric constants of polyimides could be reduced by introducing fluorine [5,6,7,8], aliphatic/alicyclic structure [9,10,11], large side groups [12,13,14,15,16] or air [17,18,19,20] into polymer backbones. Zhi et al. [21] synthesized a series of semi-aromatic polyimide sheets, which were derived from the copolymerization of an alicyclic tetrahedral dianhydride (HBPDA) and an aromatic diamine mixture containing three flexible diamines. The copolyimide films showed dielectric constant of 2.56 and dielectric loss of 0.004 at 1 MHz. However, the glass transition temperatures of the films were lower than 290 °C and no mechanical properties were described. Ma et al. [22] prepared porous low-dielectric polyimide films by using microemulsion method, in which water droplets acted as template. Polyhedral oligomeric polysiloxane (POSS) with hierarchical porous structure was coated on both sides of planar polyimide (PI) films to prepare sandwich composite films. The films exhibited ultra-low dielectric constant (2.28) and dielectric loss (0.005) at 1 MHz, as well as low water absorption (0.56%). However, the composite films were brittle, having elongation at breakage of lower than 10%, and no dielectric properties at high frequencies were described.

Compared with dielectric constant, there are few reports on the reducing of dielectric loss of polyimide films at high frequencies (>10 GHz). Under the alternating electric filed, the dipole of the polymer chain is deflected back and forth. Dielectric loss mainly comes from the energy loss generated by the dipole in order to overcome the friction resistance and dissipated in the form of heat energy. Hence, it is reasonable to consider that the dipole deflection amplitude in alternating electric field can be limited by decreasing of the molecular chain spacing and increasing of the interchain interaction force, thus resulting in the low dielectric loss.

Fluorinated aromatic polyimides have been reported to exhibit low dielectric constant and low dissipation factor [23]. However, they have usually shown low modulus and high coefficient of thermal expansion, and are not suitable to be the base film of flexible printed circuit board. In this study, fluorinated aromatic polyimide films with high modulus and excellent dielectric properties at high frequency were prepared by introducingrigid rod-like structures in polymer backbone. Influence of the chemical structures on the combined properties of these films were systematically investigated. The relationship between the tight packing of polymer chains and the dielectric properties at high frequency was further illustrated by molecular dynamics simulation.

## 2. Materials and Methods

### 2.1. Materials

4,4′-(Hexafluoroisopropylidene) diphthalic anhydride (6FDA), 2,2′-Bis(trifluoro-methyl)benzidine (TFDB) and 3,3′,4,4′-Biphenyltetracarboxylic dianhydride (BPDA) was purchased from Chinatech (Tianjin, China) Chemical Co., Ltd. and dianhydrides including 6FDA and BPDA were used after vacuum drying at 160 °C for 3 h. Dimethylacetamide (DMAc) was purchased from Concord Technology (Tianjin, China) Co., Ltd. and used without distilled. Acetic anhydride was obtained from Sinopharm Chemical Reagent Co., Ltd. (Shanghai, China) while isoquinoline was obtained from Shanghai Aladdin Biochemical Technology Co., Ltd., (Shanghai, China) and both of them were directly used.

### 2.2. Methods

**Scheme 1:**
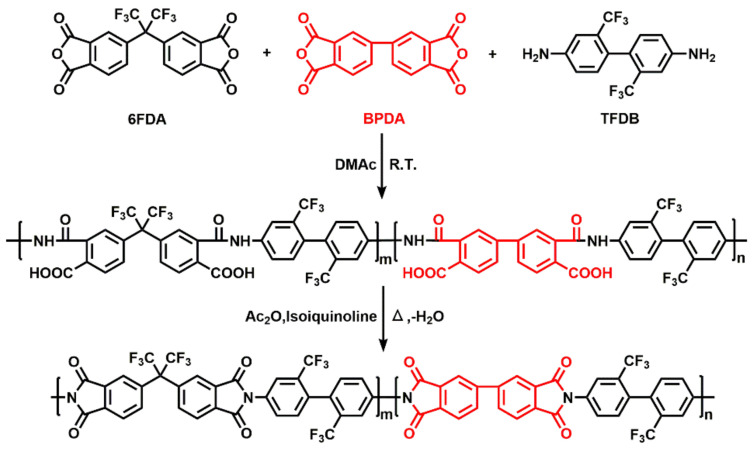
Synthesis of the fluorinated aromatic polyimide films.

Fluorinated aromatic polyimide films were prepared by a two-step chemical imidization method displayed in Scheme 1. 32.02 g (100 mmol) of TFDB and 282 mL of DMAc were added into a three-neck round-bottom flask under a nitrogen atmosphere. After TFDB was completely dissolved, 13.33 g (30 mmol) of 6FDA and 20.60 g (70 mmol) of BPDA were added separately into the solution. The solution was mechanically stirred at room temperature for 24 h to give a homogeneous poly (amic acid) (PAA) solution with a solid content of 20% (FAPI-70).

The PAA solution, diluted by DMAc to 15% solid content, was mixed with a chemical imidization reagent (acetic anhydride: isoquinoline = 1:1, molar ratio), and then defoamed completely by a de-aerating mixer. The mixture was cast onto a dust-free glass plate with an adjustable doctor blade, and was then thermally baked in an oven with the following procedure: 65 °C/5 min, 75 °C/5 min, 85 °C/15 min. After most of the solvent was evaporated, a partially-cured film was peeled off from the glass plate, which was then fixed on the metal fixture and imidized completely in an oven with the following procedure to give the fluorinated aromatic polyimide (FAPI-70) film: 180 °C/5 min, 210 °C/3 min, 330 °C/3 min.

Similarly, a series of FAPI films with different molar ratios of BPDA/6FDA, including FAPI-0 (BPDA/6FDA = 0/100), FAPI-80(BPDA/6FDA = 80/20), FAPI-90(BPDA/6FDA = 90/10), FAPI-100(BPDA/6FDA = 100/0), have been prepared, successively.

### 2.3. Characterization

Fourier transform infrared spectroscopy (FTIR) were carried out on TENSOR27 of Bruker Company with scanning wavenumber ranging from 3400 cm^−1^ to 600 cm^−1^ at Attenuated Total Reflection (ATR) mode to measure the imidization degree.

^13^C NMR spectra were performed on a Bruker solid state NMR spectrometer AVANCE III 400 and collected at different spinning speeds to locating the chemical shift of spinning side bands.

Dielectric properties of FAPI films were measured on N5227B PNA Network Analyzer of Keysight Company equipped with cylindrical cavity resonance clamps at high frequencies of 10 GHz, 24 GHz, 40 GHz and 60 GHz, respectively.

Wide-angle X-ray diffraction (WXRD) were executed in the range of 5°–50° at a scanning speed of 5 (°)·min^−1^ on the Empyrean diffractometer of Malvern Panalytical Company with Cu Kα radiation (λ = 0.154 nm) at 40 kV.

Coefficients of thermal expansion (*CTE*) of FAPI films were undertaken on the Q400 thermomechanical analyzer with a heating rate of 5 °C·min^−1^ and a load force of 0.05 N under a nitrogen flow. Before testing, the samples were treated at 150 °C for 5 min to remove the residual stress.

Birefringence index of FAPI films were measured by a prism coupler (Metric model PC-2000, Korea) at a wavelength of 633 nm.

Mechanical properties were measured on 5567 universal testing machines of Instron Company at a drawing rate of 2.0 mm·min^−1^.

Thermogravimetric analysis (TGA) was performed on a TA Q50 thermal analyzer in the range of 40 °C–800 °C with a heating rate of 20 °C·min^−1^ in nitrogen and air, respectively. The sample was preheated to 150 °C for 5 min to remove moisture prior to testing.

Dynamic mechanical analysis (DMA) was performed on a TA Q800 thermal analyzer in the range of 40 °C–450 °C with a heating rate of 5 °C·min^−1^ and a load frequency of 1 Hz under nitrogen atmosphere.

Ultraviolet-visible (UV-Vis) spectra of the FAPI films were recorded on a Hitachi U3900 spectrophotometer from 180 nm to 800 nm at room temperature with a sample’s thickness of 25 μm. The yellowness and haze of the FAPI films were evaluated by X-rite Color i7 spectrophotometer, using an observational angle of 8° and a CIE (Commission International de I’Eclairage) standard D65 illuminant.

### 2.4. Computational Details

Molecular dynamics simulation was performed using Forcite module as implemented in Materials Studio 2019 software package. The molecular structures of FAPI films were constructed and their relevant parameters were calculated according to the following procedure:

Firstly, the repeating units of polyimides were built, and the single molecular chains containing 20 repeating units of two homopolymers (FAPI-0 and FAPI-100) were established by the “homopolymer” tool [24,25] and followed by geometry optimization. Then, the amorphous polymeric models of FAPI-0 and FAPI-100 were constructed with 10 optimized chains using Amorphous Cell module. It’s testified that reasonable molecular chain length and quantities are critical to simulate the real model, i.e., a short chain length can lead to end effects, whereas a long chain leads to increased simulation difficulties [26]. As for the random polymers, 10 molecular chains and 20 repeating units were adopted as well to the amorphous polymeric models with different probabilities on the basis of the copolymerization proportion of dianhydride monomers. All of the amorphous polymeric models were optimized ulteriorly to avoid convergence failure.

Molecular dynamics simulation was employed on the polyimide models to acquire the final equilibrium structures. As the initial polymeric models with the density of 0.5 g/cm^3^, a compressing procedure was carried out at 0.5 GPa under NPT ensemble. Afterwards, the frame with the closest density to the measured sample was exported for the NVT annealing dynamics with 10 cycles from 850 to 400 K to minimize the undesired inner stress between the chains. Then, the frame with the lowest potential energy was selected to run the final equilibration process under NVT ensemble at 0.0001 GPa and 298K for 500 ps. COMPASS II force field was utilized in the MD simulations. The Ewald method with an accuracy of 0.0001 kcal/mol was used to calculate both the non-bonded Van der Waals interactions and the non-bonded long-range electrostatic interactions. Furthermore, the Nosé thermostat and the Berendsen barostat were used to control the temperature and pressure at a time step of 1 fs, respectively.

Based on the final equilibrated PI models, Radius of gyration (*R*_g_), Kuhn segment length (*l*_b_), Cohesive Energy Density (*CED*) and Fractional Free Volume (*FFV*) were calculated.

## 3. Results and Discussion

### 3.1. Characterization

**Figure 1:**
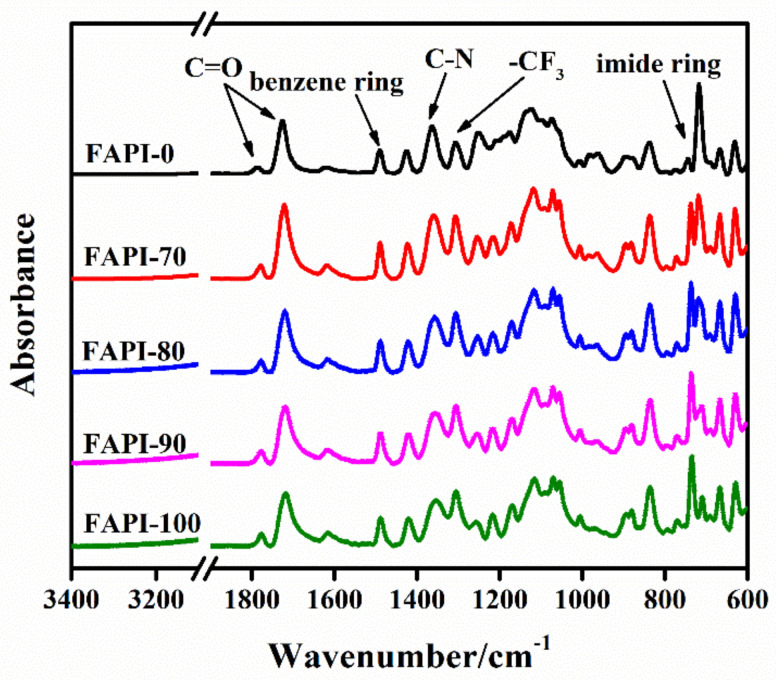
ATR-FTIR spectra of the FAPI films with different biphenyl group content in polymer backbone.

A series of fluorinated aromatic polyimides with different biphenyl group content in polymer backbone by changing the molar ratios of 6FDA/BPDA were successfully synthesized by chemical imidization method. Compared with the traditional thermal imidization method, chemical imidization method can initiate the imidization of PAA to polyimide at lower temperature. It is proved that the imidization procedure has been completed with the advent of 1778 cm^−1^ (C=O asymmetrical stretching of imide groups), 1720 cm^−1^ (C=O symmetrical stretching of imide groups), 1358 cm^−1^ (C−N stretching) and 737 cm^−1^ (C=O bending of imide rings) (Figure 1). No signal of −OH in −COOH (3400–2500 cm^−1^, a wide and uneven peak) was detected, implying that chemical imidization is a more effective pathway than thermal imidization.

**Figure 2:**
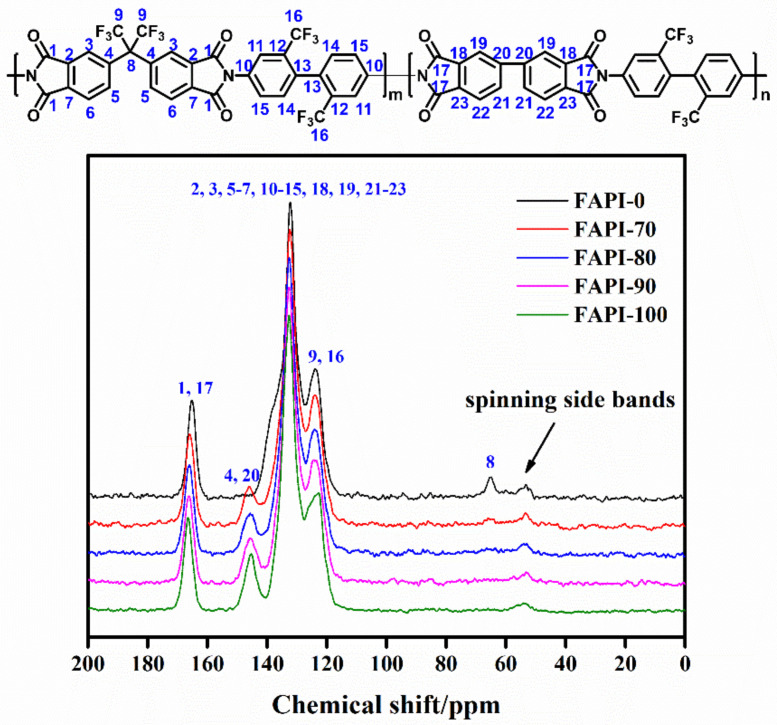
^13^C solid state NMR spectra of the FAPI films with different biphenyl group content in polymer backbone.

Figure 2 depicts ^13^C solid state NMR (SSNMR) spectra of the FAPI films. The peaks of ^13^C in a variety of chemical environments have been detected, including the peaks at 166 ppm for C=O, 146 and 132 ppm for benzene rings, 124 ppm for −CF_3_ and 65 ppm for the quaternary carbon atom of −C(CF_3_)_2_ group, respectively. The peak at 146 ppm is assigned to the carbon atoms which connected the benzene rings in the biphenyl unit. With increasing of biphenyl contents in the polymer backbones, the peak intensities became more and more obvious, meanwhile the intensity of −C(CF_3_)_2_ at 65 ppm decreased. The peak of the quaternary carbon atom of −C(CF_3_)_2_ group is not so visible due to the weak Nuclear Overhauser Effect (NOE) compared with other carbon atoms.

### 3.2. Mechanical and Optical Properties

**Figure 3:**
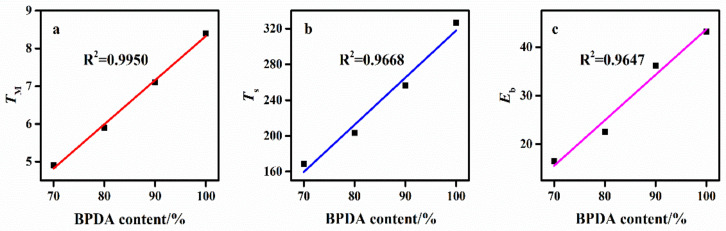
Relationship between mechanical properties and the biphenyl group content in polymer backbone.

Table 1 depicts the mechanical properties of the FAPI films including tensile modulus (*T*_M_), tensile strength (*T*_s_) and elongation at breakage (*E*_b_). With the introduction of rigid rod-like biphenyl groups in the polyimide backbones, the FAPI films exhibited enhanced mechanical properties (Figure 3). It can be seen that both film modulus and strength have been improved gradually with increasing of the biphenyl contents in polymer backbones. For instance, FAPI-100 showed the highest *T*_M_ (8.4 GPa) and *T*_s_ (326.7MPa), compared with FAPI-80 (*T*_M_ = 5.9 GPa, *T*_s_ = 203.3 MPa) and FAPI-70 (*T*_M_ = 4.9 GPa, *T*_s_ = 168.5 MPa), respectively. The elongation at breakages was also enhanced by increasing the biphenyl group contents in polymer backbone from 16.5% (FAPI-70) to 43.2% (FAPI-100), probably due to the higher polycondensation reactivity of the biphenyl-containing dianhydride monomer.

**Figure 4:**
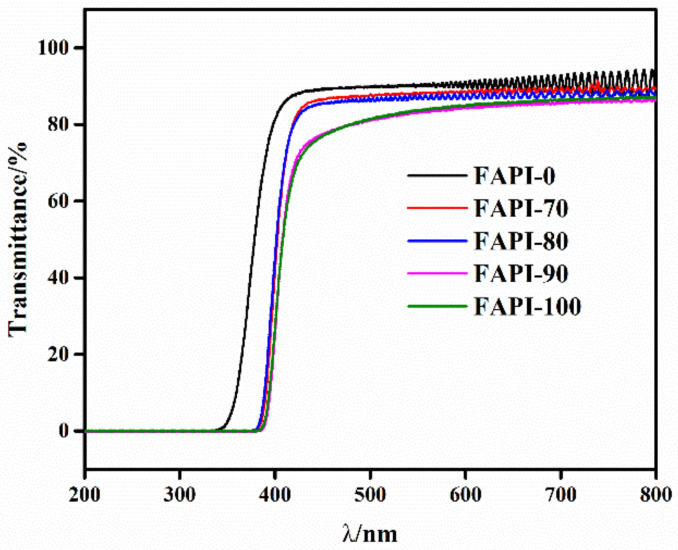
Ultraviolet-visible (UV-vis) spectra of the FAPI films with different biphenyl content in polymer backbone.

Figure 4 shows the UV spectra of the FAPI films, which exhibits great visible transparence. With the increasing of the biphenyl contents in polyimide backbones, the light transmittance at 500 nm (*T*_500_) was reduced gradually from 89.7% (FAPI-0) to 81.4% (FAPI-100) (Table 2), and the cutoff wavelength (*λ_cut_*) was red-shifted from 334 nm (FAPI-0) to 381 nm (FAPI-100). Moreover, the yellowness (*YI*) increased from 1.3 (FAPI-0) to 6.9 (FAPI-100) and haze (*HZ*) from 1.7 (FAPI-0) to 4.1 (FAPI-100), respectively. Clearly, the optical transparency of FAPI films was deteriorated with the increasing of the biphenyl contents in polymer backbone, probably attributed to the higher bandgap energy of the biphenyl groups than the −CF_3_ groups in polymer backbones.

### 3.3. Thermal Properties

**Figure 5:**
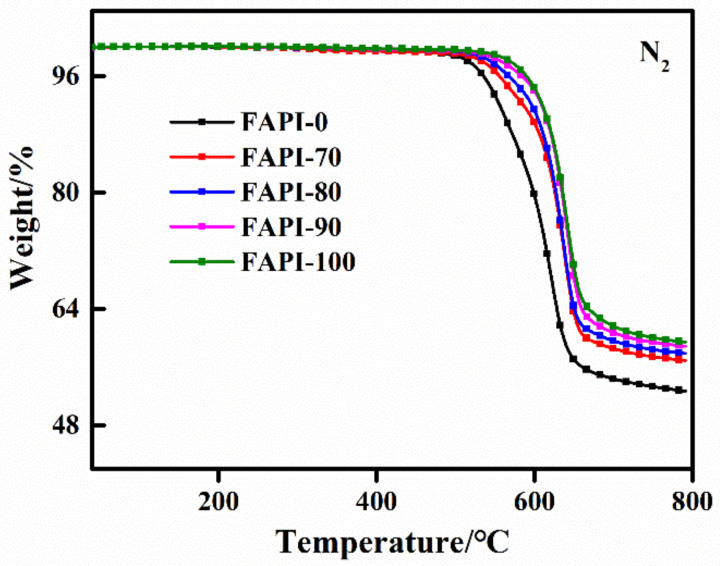
TGA curves of the FAPI films with different biphenyl content in polymer backbone.

Figure 5 compares TGA curves of the FAPI films with different biphenyl contents in polymer backbones, and the corresponding thermal decomposition temperature (*T*_d_) and glass transition temperature (*T*_g_) are summarized in Table 3. The FAPI films showed great thermostability both in air and nitrogen. Moreover, no obvious weight loss was detected until the temperature was scanned up to 500 °C, implying that the imidization process was successfully completed and no residue of organic volatile existed in the films. As the −CF_3_ groups are easily decomposed at high temperature, the *T*_d_ and *R*_750_ were found to increase gradually with the decreasing of −CF_3_ groups in the polyimide backbones. The *T*_d5_ and *T*_d10_ were measured at 541.8 °C and 564.3 °C (FAPI-0), respectively, about 52–55 °C lower than that of FAPI-100 (*T*_d5_ = 596.7 °C, *T*_d10_ = 616.7 °C). At 750 °C, the FAPI films showed residual weights of 53.3 to 60.1%.

**Figure 6:**
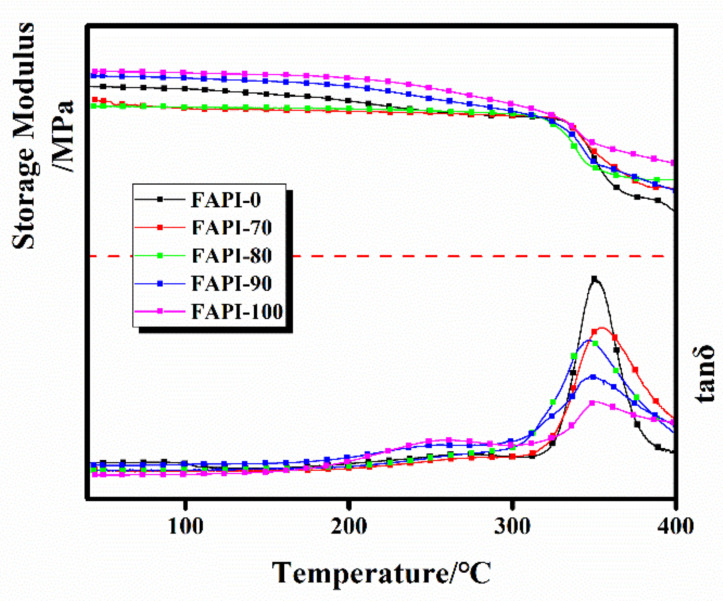
DMA curves of the FAPI films with different biphenyl content in polymer backbone.

The glass transition temperature of the FAPI films were measured at about 350 °C by DMA (Figure 6). The *T*_g_ values of the FAPI films were almost identical although the biphenyl contents varied. This could be attributed to the steric hindrance caused by the bulky −CF_3_ groups linked at the biphenyl groups, which obstructed the twist moving of the polymer backbone and resulted in the high glass transition temperature. It is worth noting that all of the FAPI films showed *T*_g_ values which are higher than the finally cured temperature (330 °C), indicating that the imidization process is a local process of cyclization and dehydration without much molecular chain moving ability [27].

### 3.4. Dielectric Properties

**Figure 7:**
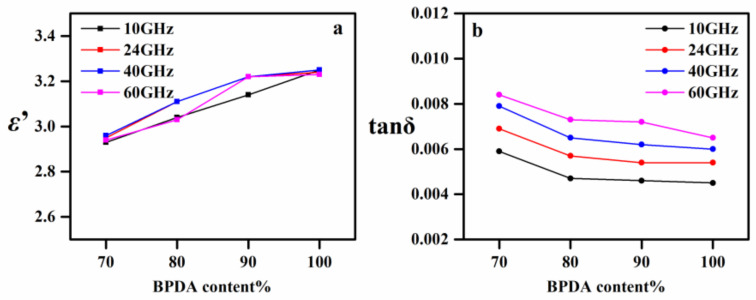
Dielectric constant (**a**) and dissipation factor (**b**) of the FAPI films at different high frequencies (10–60 GHz).

Dielectric properties of the FAPI films were measured on Network Analyzer of Keysight Company equipped with cylindrical cavity resonance clamps at high frequencies from 10 to 60 GHz, respectively (Table 4). Two interesting phenomena were attracted our attention. Compared with the dielectric properties of different films at one constant frequency, such as at 10 GHz, the dielectric constant (*ε*’) increased gradually with the increasing of the biphenyl contents in polyimide backbones, while the dielectric dissipation factor (*tanδ*) decreased (Figure 7). Moreover, if comparing the same film at different frequency, the dielectric constant does not change obviously, but the *tanδ* was increased about 42–56% from 10 to 60 GHz (Figure 7b). For instance, compared to both FAPI-80 and FAPI-90, FAPI-70 has a lower *ε*’ (2.93) and higher *tanδ* (5.9 × 10^−3^) at 10 GHz. When the frequency increased from 10 GHz to 60 GHz, it’s *ε*’ values kept almost constant, but the *tanδ* increased from 5.9 × 10^−3^ to 8.4 × 10^−3^.

Introduction of −CF_3_ groups in polymer backbones has been considered as an effective way to reduce the dielectric constant of polyimides. This is attributed to the strong electron absorption and large free volume of fluorinated groups. It can be seen that the dielectric constants of the FAPI films were changed from 2.68 (FAPI-0) to 3.25 (FAPI-100) at 10 GHz, which is closely related to the decline of the fluorine contents (*F*%). The frequency also affects the dielectric constant. Under the applied electric field, dielectric properties of a polymer are mainly attributed by three kinds of polarization ways, namely orientation polarization, atomic polarization and electron polarization with the inherent frequencies of 10^9^ Hz (1 GHz), 10^12^ Hz (10^3^ GHz) and 10^15^ Hz (10^6^ GHz), respectively [28]. The atomic polarization and electron polarization are also called deformation polarizations. In our research, the test frequencies (10–60 GHz) are dominantly located in the range of inherent frequencies of orientation polarization and atomic polarization. If the inherent frequency of orientation polarization of the FAPI films is lower than the frequency of the applied electric field, the dielectric constants of different testing samples would not change much. Furthermore, because the frequency of the applied electric field does not reach the inherent frequency of deformation polarization, the polarization of atoms and electrons has sufficient relaxation time, having no influence on dielectric constant (*ε*’).

The dielectric loss of polymers originates from the energy loss generated to overcome intramolecular friction when dipoles, atoms and electrons are deflected in an alternating electric field. From this point of view, the internal factors affecting dielectric loss mainly lie in the number of dipoles and the deflection amplitude of dipoles. The more the dipoles and the greater the amplitude of deflection, the higher the dielectric loss. As shown in Table 4, the dielectric loss of FAPI films always decreases with the increase of the biphenyl content at each frequency, that means the introduction of rigid groups does help to weaken the deflection of dipoles. On the other hand, as the frequency was changed from 10 GHz to 60 GHz, the dielectric loss increased gradually. This could be interpreted that the frequency of atomic deflection increased with the increasing of test frequency, which intensified the internal friction between molecules.

Beside the structural factors which would affect the dielectric properties of a polymer, the water absorption is another key factor. The water absorption (*W*_a_) of the FAPI films was measured in the range of 0.25–0.40% (Table 4). With the introduction of rigid biphenyl units, the *W*_a_ of the FAPI films are slightly improved on account of the reduction of *F*%. At high frequencies, the influence of water molecules on dielectric loss cannot be ignored. Compared with polymer, water molecules with strong polarity are deflected more sharply under the alternating electric field, which is also positively correlated with frequency.

### 3.5. Effect of Polymer Chain Orientation on Dielectric Properties

**Figure 8:**
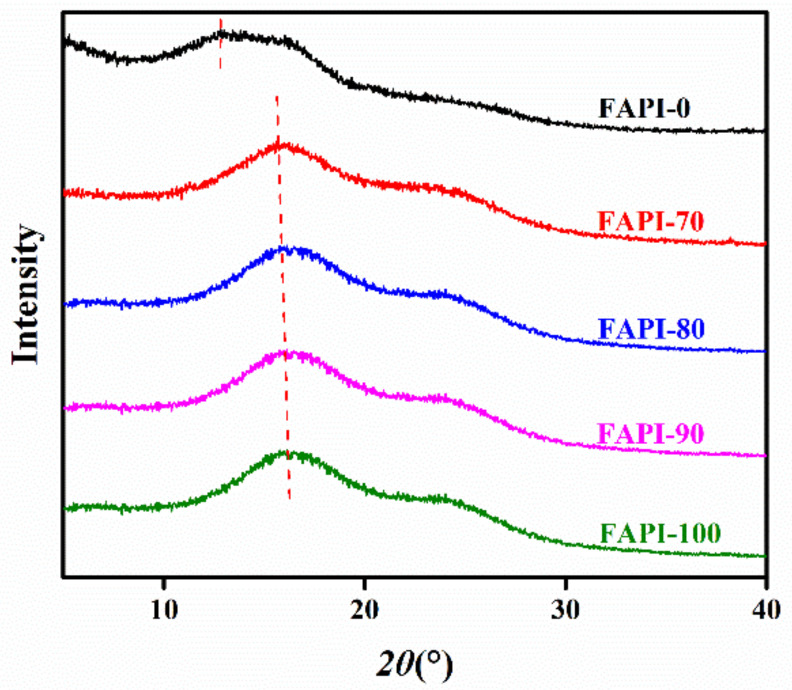
WXRD curves of the FAPI films with different biphenyl content in polymer backbone.

Figure 8 compares the WXRD spectra of the FAPI films. The crystal plane diffraction angle (*2θ*) of the FAPI films were gradually increased with the increasing of the biphenyl contents in polymer backbones (Table 5). As a consequence, the polymer chain spacing (*d*) decreased gradually from 6.7 Å to 4.8 Å according to the Bragg equation, indicating that the introduction of biphenyl groups in polyimide backbones has effectively enhanced the polymer chain orientation and the inter-chain interactions, and thus increased the polymer chain packing density. The strong interchain interaction limited the deflection of the dipole, atoms and electrons in the electric field, resulting in a reduction of the energy consumed to overcome the friction in the polymer chains, thus lowering the dielectric loss.

**Figure 9:**
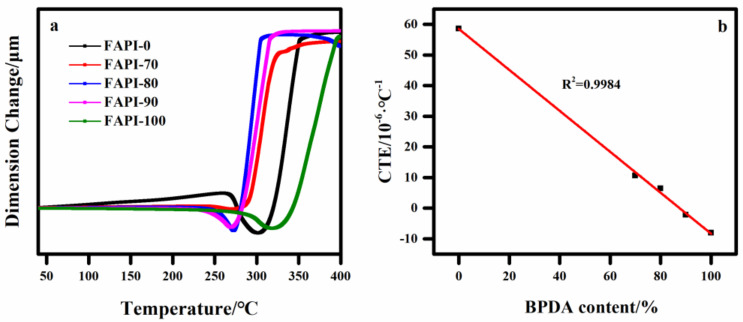
(**a**) TMA curve of the FAPI films; (**b**) The relationship between *CTE* and biphenyl content in polymer backbone.

Figure 9 compares the TMA curves (a) and *CTE* values (b) of the FAPI films. With the increasing of biphenyl content and the decreasing of −CF_3_ groups in polyimide backbone, the *CTE* value of the FAPI films was decreased significantly from 58.6 × 10^−6^/°C (FAPI-0) to 10.6 × 10^−6^/°C (FAPI-70), and 6.4 × 10^−6^/°C (FAPI-80), respectively, which is probably attributed to the reduction of the free volume of polymer structure [29]. The rigid polyimide backbones showed weaker in-plane expansion behavior and stronger in-plane orientation extent, resulted in low *CTE*. Surprisingly, the FAPI-90 and FAPI-100 showed a negative *CTE* values, implying that the polymer aggregate was shrank at high temperature.

**Figure 10:**
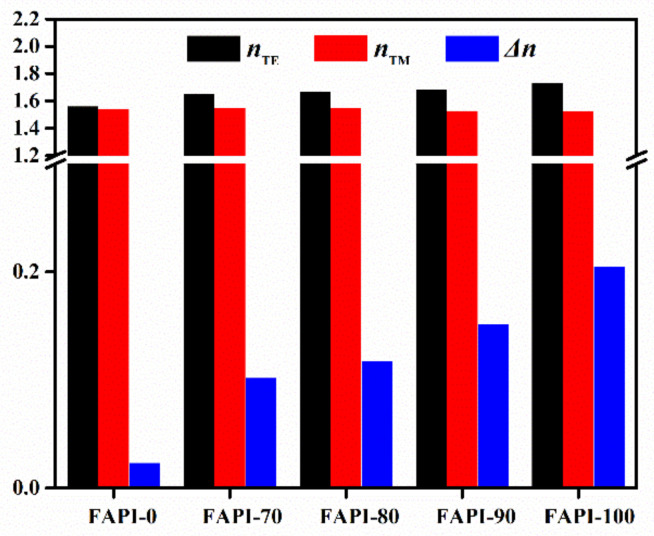
Birefringence index of the FAPI films with different biphenyl content in polymer backbone.

Figure 10 compares the birefringence behaviors of the FAPI films measured by a prism coupler at a wavelength of 633 nm. With the increasing of biphenyl contents in polymer backbone, the in-plane refractive index (*n*_TE_) of the FAPI films were increased gradually from 1.5613 (FAPI-0) to 1.7303 (FAPI-100), and the out-of-plane refractive index (*n*_TM_) were reduced accordingly from 1.5379 (FAPI-0) to 1.5250 (FAPI-100). Thus, the *Δn* value was increased from 0.0234 (FAPI-0) to 0.2053 (FAPI-100), indicating that the polymer orientation extent was also increased gradually. The results further proved that the dielectric loss of the FAPI films at high frequencies could be reduced by enhancing the polymer chain orientation and stacking density, which limited the deflection of the dipole, atom, electron under alternating electric field.

### 3.6. Molecular Simulation

Molecular Dynamics (MD) simulation was employed to demonstrate the above conclusion from a microscopic point of view. Firstly, a series of theoretical models of the FAPI films which are closed to the real state were established by validating appropriate parameters and conditions. Chain Rigidity, Intermolecular Force and Chain Stacking were then investigated by calculating the corresponding parameters.

#### 3.6.1. Chain Rigidity

Radius of Gyration (*R*_g_) and Kuhn segment length (*l*_b_) were utilized to reflect the flexibility of molecular chains of studied PIs. *R*_g_ is defined as the distance from the point of concentration assumed by the differential mass of the object to the axis of rotation [30], and *l*_b_ is defined as the segment length of the polymer chain regarded as a free-connected chain with segments as statistical units. In general, the lower the *R*_g_ and *l*_b_, the better the chain’s flexibility. Table 6 compares the *R*_g_ and *l*_b_ values of the FAPI films with different biphenyl groups in the polyimide backbones. With the increasing of rigid rod-like biphenyl structures, *R*_g_ and *l*_b_ values were increased gradually from 41.26 (FAPI-0) to 56.42 (FAPI-90) except for FAPI-100, indicating that the rigid rod-like segments ensured the polymer chain conformation being more straightened and less twisted and tangled.

#### 3.6.2. Intermolecular Force

In order to better quantify the intermolecular forces, Cohesive Energy Density (*CED*) was calculated through the established models. *CED* is the energy required to overcome the intermolecular force of vaporization of 1 mol of aggregates per unit volume [31], which means that the aggregate structures with higher *CED* usually have stronger intermolecular forces. By replacing the −CF_3_ group with biphenyl groups in the polyimide backbones, the *CED* of the FAPI films were increased from 3.13 (FAPI-0) to 4.34 (FAPI-100), testifying that the rigid-like biphenyl segments have effectively enhanced the intermolecular force and resulted in the reducing of the dipole deflection in the electric field.

#### 3.6.3. Chain Stacking

**Figure 11:**
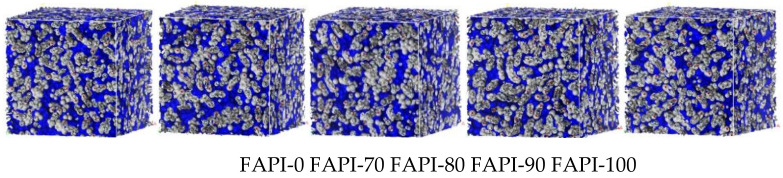
3D periodic boundary cell of the FAPI films (gray part represents the molecule chain skeleton while blue part represents the free volume).

In general, total volume (*V*) of a polymer can be divided into two parts, one part is occupied by atoms (*V*_W_), the rest is the free volume. The fractional free volume (*FFV*) is the fraction of the free volume to the total volume, including the random distribution of unoccupied holes in polymer aggregate phase (static) and the space formed as the density of the material fluctuates and the molecules move (dynamic) [32], which represents the stacking degree of macromolecular chains. The *FFV* of the FAPI films reduced from 23.61% (FAPI-0) to 13.65% (FAPI-100) with the increase of the biphenyl groups in the polyimide backbones as depicted in Table 4. Figure 11 compares the 3D periodic boundary cell of the FAPI films. Clearly, the aggregate structures of the FAPI films became to denser stacking with less −CF_3_ and more rigid-like biphenyl groups in the polymer backbones.

## 4. Conclusions

Fluorinated aromatic polyimide films with high modulus and excellent dielectric properties at high frequency have been prepared. The polyimide films exhibited great combined mechanical, thermal and colorless transparence properties, as well as dielectric properties, including tensile modulus of up to 8.4 GPa, strength of up to 326.7 MPa, glass transition temperature (*T*_g_) of 346.3–351.6 °C, thermal decomposition temperature in air (*T*_d5_) of 541.8–596.7 °C, as well as high colorless transmittance over 81.2% at 500 nm. Moreover, the polyimide films also showed dielectric constant of 2.68–3.25 which did not change obviously at frequency from 10 GHz to 60 GHz, and dielectric loss as low as 0.0045 at 10 GHz which is suitable for signal transportation applications at high frequencies. The relationship of molecular structure and dielectric loss was estimated by molecular dynamics simulations.

## Figures and Tables

**Table 1:** Mechanical properties of the FAPI films.

FAPI Films	*T*_M_/GPa	*T*_s_/MPa	*E*_b_/%	Thickness/μm
FAPI-0	3.1	99.4	5.8	65.5
FAPI-70	4.9	168.5	16.5	55.0
FAPI-80	5.9	203.3	22.5	47.5
FAPI-90	7.1	256.4	36.2	48.0
FAPI-100	8.4	326.7	43.2	50.0

**Table 2:** Optical data of the FAPI films.

FAPI Films	*T*_500_ ^a^/%	*λ*_cut_ ^b^/nm	*YI* ^c^	*HZ* ^d^
FAPI-0	89.7	334	1.3	1.7
FAPI-70	87.7	379	2.6	1.3
FAPI-80	86.1	376	3.3	1.2
FAPI-90	81.2	381	6.4	4.7
FAPI-100	81.4	381	6.9	4.1

^a^ Transmittance at 500 nm; ^b^ Cutoff wavelength; ^c^ Yellowness; ^d^ Haze.

**Table 3:** Thermal properties of the FAPI films.

FAPI Films	*T*_g_/°C	*T*_d5_^a^/°C	*T*_d10_^b^/°C	*R*_750_^c^/%
FAPI-0	351.0	541.8	564.3	53.3
FAPI-70	354.2	563.8	598.0	57.5
FAPI-80	346.3	576.7	605.0	58.4
FAPI-90	348.4	593.4	616.0	59.4
FAPI-100	351.6	596.7	616.7	60.1

^a^ Decomposition temperature at 5% weight loss; ^b^ Decomposition temperature at 10% weight loss; ^c^ Residual weight at 750 °C.

**Table 4:** Dielectric properties of the FAPI films at high frequencies.

FAPI Films	10 GHz		24 GHz		40 GHz		60 GHz		*F*%	*W*_a_/%
	*ε*’	*tanδ*	*ε*’	*tanδ*	*ε*’	*tanδ*	*ε*’	*tanδ*		
FAPI-0	2.68	0.0059	2.73	0.0067	2.72	0.0076	2.70	0.0087	31.3	0.25
FAPI-70	2.93	0.0059	2.95	0.0069	2.96	0.0079	2.94	0.0084	23.2	0.30
FAPI-80	3.04	0.0047	3.11	0.0057	3.11	0.0065	3.03	0.0073	22.0	0.21
FAPI-90	3.14	0.0046	3.22	0.0054	3.22	0.0062	3.22	0.0072	20.8	0.40
FAPI-100	3.25	0.0045	3.24	0.0054	3.25	0.0060	3.23	0.0065	19.7	0.37

**Table 5:** WXRD, TMA and BR data of the FAPI films.

FAPI Films	*2θ*/°	*d*/Å	*n*_TE_	*n*_TM_	Δ*n*
FAPI-0	13.2	6.7	1.5613	1.5379	0.0234
FAPI-70	17.0	5.5	1.6543	1.5523	0.1020
FAPI-80	17.1	5.4	1.6669	1.5496	0.1173
FAPI-90	17.6	5.1	1.6813	1.5294	0.1518
FAPI-100	17.9	4.8	1.7303	1.5250	0.2053

**Table 6:** Computational results of the FAPI films.

FAPI Films	*R*_g_/Å	*l*_b_/Å	*CED*/ ×10^8^ J·m^−3^	*V*^a^/Å^3^	*V*_W_^b^/Å^3^	*FFV* ^c^
FAPI-0	41.26	23.85	3.13	171,000	100,477	23.61%
FAPI-70	49.06	35.05	3.56	141,940	89,624	17.91%
FAPI-80	53.31	42.44	4.25	137,822	88,157	16.85%
FAPI-90	56.42	50.47	3.92	134,362	86,610	16.20%
FAPI-100	51.40	46.76	4.34	127,904	84,962	13.65%

^a^ Total volume; ^b^ Van der Waals volume; ^c^ Fractional free volume, *FFV* = (*V* − 1.3*V*_W_)/*V.*

## Data Availability

The data presented in this study are available on request from the corresponding author.
